# Stakeholders’ perspectives on addressing climate change and respiratory health impacts in Nigeria

**DOI:** 10.11604/pamj.2025.51.14.47414

**Published:** 2025-05-14

**Authors:** Olayemi Oluseun Akinnola, Alexander Iseolorunkanmi, Faatihah Adeyinka Niyi-Odumosu, Temitope Eyitayo Akinnola, Boni Maxime Ale, Davies Adeloye, Obianuju Beatrice Ozoh

**Affiliations:** 1 Department of Biological Sciences, Covenant University, Ota, Nigeria,; 2 Covenant University Medical Centre, Covenant University, Ota, Nigeria,; 3 School of Applied Sciences, University of the West of England, Bristol, United Kingdom,; 4 Department of Industrial Relations and Human Resource Management, Lagos State University, Ojo, Nigeria,; 5 Cardiovascular Research Unit, University of Abuja and University of Abuja Teaching Hospital, Gwagwalada Abuja, Nigeria,; 6 Institute of Tropical and Infectious Diseases, University of Nairobi, Nairobi, Kenya,; 7 Holo Global Health Research Institute, Nairobi, Kenya; 8 School of Public Health, Moi University, Nairobi, Kenya,; 9 School of Health and Life Sciences, Teesside University, Middlesbrough, United Kingdom,; 10 Department of Medicine, Faculty of Clinical Sciences, College of Medicine, University of Lagos, Lagos, Nigeria,; 11 Lagos University Teaching Hospital Idi-Araba, Lagos, Nigeria

**Keywords:** Climate change, health, Lagos, Nigeria, stakeholders, vulnerable populations, policy

## Abstract

The impacts of climate change on respiratory health are increasingly becoming a significant challenge in Nigerian cities, particularly in Lagos and Ogun States. Engaging stakeholders in discussions about climate change and health is crucial for addressing these challenges. The climate change and respiratory health (C2Rest) Nigeria Study team facilitated a stakeholder engagement to discuss climate change and health impacts in Nigeria, aiming to explore feasible solutions relevant to research, policy, and practice. A stakeholder meeting was conducted on April 23^rd^, 2024, involving participants from Lagos and Ogun States. The framework for the activities was adapted from Gardner´s climate change engagement pathways, which examine key drivers (8 questions), barriers (7 questions), and adaptation pathways (3 questions). Data were collected through note-taking, written contributions, audio and video recording, and subsequently transcribed and analysed thematically. A total of 42 registered participants, comprising government officials, academicians, healthcare professionals, community and religious leaders, attended the stakeholder engagement meeting. Participants made group presentations and submitted a total of 380 written contributions. There were varying views on the drivers of climate change, but there was consensus that the most vulnerable populations include the poor, homeless, pregnant women, children, the elderly, and individuals with underlying health conditions. An important barrier identified was the sociocultural belief that climate change is an act of God or spirits. Financial constraints were a recurrent theme in discussions about mitigation and adaptation to climate change. This report provides valuable information on the most vulnerable population to the effects of climate change in Nigeria and identifies sociocultural beliefs and financial constraints as key barriers to the adoption of effective mitigation and adaptation behaviors.

## Introduction

Climate change, characterized by significant alterations in temperature, precipitation patterns, and increased frequency of extreme weather events, poses a substantial threat to global health [1]. Among the numerous health impacts, respiratory health is particularly vulnerable to climate-related changes [2]. Nigeria, particularly in urban areas such as Lagos and Ogun States, experiences compounded challenges due to rapid urbanization, industrial activities, and high population density [3]. These factors worsen the effects of climate change on respiratory health through increased exposure to air pollutants, heightened allergen and mould levels, and more efficient spread of respiratory infections [4,5]. Increased levels of ground-level ozone from climate change can lead to respiratory problems such as chronic obstructive pulmonary disease (COPD), asthma, and asthma exacerbations [6,7]. Higher environmental temperatures exacerbate COPD, extend the allergenicity of pollen, and promote mould growth in homes and workplaces, worsening symptoms of asthma and allergic rhinitis [8-10].

While the environmental impacts of climate change are evident in extreme weather events, severe heat waves, loss of agricultural land, and consequent devastation of livelihoods, the link between climate change and respiratory health, particularly in vulnerable communities in low-income to middle-income countries (LMICs) such as Nigeria, has not been well described and consequently not prioritized [11-14].

Engaging stakeholders is essential for developing effective strategies to mitigate and adapt to the health impacts of climate change [15]. Involves government agencies that are tasked with the responsibility of developing and implementing climate change mitigation and adaptation strategies, healthcare professionals who are facing increasing climate-related illnesses and whose practices may also contribute to greenhouse gas emissions, community leaders who influence public opinion and behaviours, and the general public who adopt these behaviours is important as collective action is fundamental to effective implementation of climate change policies. More so, stakeholder engagement ensures that diverse perspectives and local knowledge are incorporated into policy, practice, and research agenda-setting, enhancing the relevance and acceptance of proposed interventions [15-18].

As part of the Climate Change and Respiratory (C2Rest) Nigeria Study, which aims to explore and address the impacts of climate change on respiratory health in Nigeria, we conducted a stakeholder engagement workshop aimed at developing a shared vision for mitigating and adapting to the impacts of climate change. The specific objectives were as follows: (1) to understand the causes and drivers of climate change, as well as barriers to effective climate change mitigation and adaptation from the perspectives and experiences of stakeholders; (2) to encourage collaboration with relevant stakeholders towards achieving the objectives of the C2Rest study; (3) to facilitate knowledge transfer, information sharing, and confidence building while encouraging stakeholders to become climate change advocates.

## Meeting Report

### Methods

**Stakeholder selection:** a maximum variation sampling approach was used to select key stakeholders. The goal was to have a wide representation of policymakers, climate change researchers and activists, community leaders, religious leaders, healthcare workers, and members of the community who may especially experience the effects of climate change, such as farmers and outdoor workers. We leveraged established networks and snowballing to identify participants.

**Setting and scope:** this report is based on a one-day stakeholder engagement held in Lagos State, Nigeria, on April 23^rd^, 2024. The engagement commenced with a video describing the scope of the C2Rest Nigeria Study and the need for urgent action to mitigate the effects of climate change on respiratory health. Next was a detailed presentation on the health effects of climate change and the objectives of stakeholder engagement, presented by two members of the C2Rest team (FNO and OBO) in a manner accessible to general audiences. The engagement was conducted under the guiding principles that there are “no right or wrong answers” and that “everyone´s voice and opinion mattered”. The key procedure was based on communication and inclusive engagement activities to inform, consult, and involve participants.

**Theoretical framework:** the theoretical framework (theory of change) for the engagement was adapted from Gardner *et al*. climate change engagement pathways, which explores key drivers, adaptation pathways, and barriers [19]. This framework guided the design of activities and discussions aimed at informing, consulting, and involving participants.

**Group activities and discussion plan:** a tailored communication and discussion plan was crafted for each group of stakeholders based on Gardner´s framework (Table 1). The discussion plan consisted of three main activities: drivers of climate change: this activity included eight questions and was subdivided into two aspects-initial questions and questions surrounding capacity for strategic planning. Barriers to climate change mitigation: this activity included seven questions. Adaptation pathways: this activity included three questions. Participants were divided into five small discussion groups of 6-12 members. Each group consisted of various categories of stakeholders and utilized different coloured sticky notes (neon green, pink, peach, lemon green, and orange) to record their responses, ideas, and suggestions individually. These notes were subsequently posted on a large board. Each group selected a leader to present their main ideas to the rest of the stakeholders. The open session was facilitated by FNO, who also captured and highlighted major points on a flipchart.

**Table 1:** details of group activities and questions based on Gardner *et al*.climate change engagement pathways

Activities	Questions/items
Drivers (activity 1)	What do you know about climate change and its impact?
	What is the degree of your vulnerability to climate change?
	What are your cultural or social values or beliefs regarding the causes and impact of climate change in Nigeria?
	In what capacity would you be able to mitigate the impact of climate change in Nigeria?
	Identify the vulnerable populations in your community or space.
	How do we assess the readiness of the current healthcare infrastructure?
	What are the existing strategies for mitigation and adaptation?
	What are the existing policy recommendations?
Barriers (activity 2)	What are your fears or concerns about climate change?
	Who is responsible for solving issues around climate change?
	How can we enhance public understanding of scientific consensus on climate change in the face of misinformation campaigns?
	What are the major challenges of mitigating climate change in Nigeria?
	How does media coverage influence public perceptions of climate change and contribute to scepticism or acceptance?
	What role can the media play in combating misinformation and promoting accurate information about climate change?
	How can scientists and experts build trust with communities to overcome scepticism and promote understanding of climate change?
Adaptation pathway (activity 3)	Activities around reflecting, identifying, and understanding individual or own vulnerability to climate change.
	Activities around taking responsibility to identify and develop a solution to climate change, or mitigate the impact of climate change.
	Activities to assess the level of willingness of participants to adaptation planning.

**Data collection and analysis:** data were collected through note-taking, audio and video recording, and review of the sticky notes. All the data were subsequently transcribed and thematically analyzed. The thematic analysis was deductive, guided by the Gardner theory of change [19] and focused on first identifying codes and formulating common themes and patterns in the stakeholders' contributions.

**Ethical considerations:** ethical clearance for all aspects of the C2Rest Nigeria study was obtained from the Health Research Ethics Committees of the Lagos University Teaching Hospital (HREC: 19/12/2008a) and Covenant University (CU/HREC/333/24). Additionally, social approval and clearance were obtained from the Lagos State Government (LSMH/4686/1/27) and Ogun State Government (CHREC/467/25/APP), respectively. All participants at the stakeholder engagement provided informed consent for their contributions to be shared anonymously.

**Feedback and evaluation:** at the end of the engagement meeting, participants voluntarily completed a feedback survey designed to assess their overall experience, the format and structure of the meeting, and its impact. This feedback was used to identify areas for improvement in future engagements.

## Results

**Participants:** a total of 42 registered participants took part in the stakeholder engagement meeting. These participants included government representatives (8) from the Lagos and Ogun State Ministries of Health, Lagos State Ministry of Environment and Water Resources, Lagos State Environmental Protection Agency, Ado-Odo Ota Local Government Authority and Economic Summit Group, and Mosan-Okunola Local Council Development Authority. Additionally, there were healthcare workers (12) and members of the academic community (7) from the Alimosho Local Government Area Primary Healthcare Centre, Lagos State University Teaching Hospital (LASUTH), and Lagos University Teaching Hospital (LUTH), University of Lagos, Covenant University, Bells University of Technology, and Lagos State University. Others were community members (4), environmental and climate change advocates (2), climate change-related non-profit organisations (3), members of the press (3), a traditional ruler (1), and religious leaders (2). Of the participants, 42.8% were from Lagos State, 28.5% from Ogun State, and another 28.5% did not specify their location (lived in either state).

**Written contributions:** a total of 380 written contributions were received from the five groups during the workshop sessions. The contributions per group were as follows: neon green group (80), pink group (89), peach group (62), lemon green group (74), and orange group (75).

### Key points based on the theory of change

**Drivers:** participants agreed that everyone was affected by climate change and was all vulnerable to the adverse impacts. However, there was a consensus that the most vulnerable populations to climate change were the poor, homeless, pregnant women, children, the elderly, people with pre-existing or underlying health conditions such as asthma, low-income earners, and residents of slums and flood-prone areas who cannot afford to relocate. “*Everyone is highly vulnerable due to disruption of socioeconomic development and even health*”, a participant answered.

Regarding cultural and social beliefs on the causes and impact of climate change, the stakeholders identified that most people in their communities attributed climate change events to supernatural causes, such as water spirits or acts of God, rather than human activities, and some also perceived climate change as primarily a Western issue. One participant said: “*In this part of the world, many people do not take issues of climate change seriously. They think it is a problem for the Western world, and some also believe there is nothing they can do about it, while others are overcome by the need to survive and are not interested in climate change*”.

Most participants felt they had a low individual capacity to mitigate against climate change due to poverty. The high cost of alternative cleaner fuels was identified as a factor in the continued use of fossil fuels such as charcoal and kerosene, which are commonly used in these cities. A few participants also suggested actions that could be taken by individuals and the government to mitigate the effects of climate change. For example, a participant mentioned the importance of individuals planting trees and the need for urban planning policies that segregate residential areas from industries. The statement read: “*Tree planting and separating residential areas from factories and companies is necessary*”.

The community leaders were particularly vocal about the enforcement of proper urban planning practices and the need to prevent mixed developments of residential and industrial facilities to improve air quality. They also highlighted the need for designated green zones within the communities. One traditional ruler said: “*The government should mandate that for every building erected, one tree should be planted alongside*”. The general opinion of participants was that the current healthcare infrastructure in their localities was insufficient to tackle the health impact of climate change due to the increase in the frequency of illnesses, inadequate health personnel, and diagnostic and treatment facilities. The lack of universal health coverage (UHC) was also identified to limit access to available healthcare services, and healthcare workers were also considered to be uninformed about the health effects of climate change.

A participant opined: “*Healthcare workers lack knowledge and skills in tackling health problems as a result of climate change*”, and another added, “*There is a lack of availability of minimum infrastructure requirements across all facilities. How many facilities have the minimum requirements?*” Participants not associated with government agencies were unaware of existing mitigation and adaptation strategies, implying that there is inadequate dissemination of climate change information. Those from government agencies noted funding challenges in the implementation of available strategies. A government agency representative said: “*There is already an existing ban on single-use plastics, open-air burning, waste sorting, two-bin systems, recycling, environmental education in schools, and encouraging a switch from fossil fuels to cleaner sources of energy*”. However, most participants noted that these regulations were not implemented and enforced.

**Barriers:** the participants established that everyone was directly or indirectly responsible for mitigation and adaptation to climate change. They agreed that while climate change policies and mitigation strategies are driven by the government, it was imperative for individuals to also take responsibility and engage the government to act. A participant wrote: “*Everyone is responsible; therefore, we should take the initiative and act responsibly in every space we find ourselves*”.

Key challenges in mitigating climate change in Nigeria were noted to be multifactorial, resting on individuals, the media, and the government. Low population literacy about climate change, driven by a lack of information or pervasiveness of myths about the origin of climate change, was identified as a key factor that enhanced resistance to changing personal behaviour. An interesting view was that since their ancestors cooked with fossil fuels and still lived long, there was no need to change. Furthermore, high rates of poverty coupled with cultural beliefs continue to drive biomass use. A participant said: “*It is also a critical challenge that most people believe that their ancestors used fossil fuel and lived long, so there´s nothing wrong in continuing on the same path*”.

The media's role in spreading climate change information was considered crucial, but they also agreed that personal scepticism hinders accurate delivery. One participant stated: “*They should stop encouraging false information about climate change*”. Human activities causing deforestation, along with the lack of effective governance and public cooperation, were also identified as significant barriers. Weak law enforcement and policy implementation were noted to worsen the situation. Funding constraints and corruption, lack of political will, poor institutional frameworks, and inadequate support for research institutes were also highlighted as additional barriers. The underfunding of healthcare was highlighted as an example of inadequate preparedness for climate change. A participant noted: “*Assessing the current readiness of healthcare infrastructure is a long journey, but it begins with the government allocating a higher percentage of GDP to health*”.

The group agreed that climate change researchers did not disseminate their findings in ways that were actionable for policymakers and the public. They noted that the role of researchers includes simplifying scientific information, engaging with the media, and translating the simplified information into local languages for dissemination. It was recommended that media campaigns be developed to address cultural myths and that partnerships should be established with influential figures, including community and religious leaders, to foster understanding and build trust within the communities. It was also suggested that education about climate change should begin at an early age in schools. One participant stated: ”*Climate education should be made compulsory at every level of education. This will cultivate a generation of youths who will champion and implement climate initiatives*".

**Adaptation pathways:** participants confirmed their personal vulnerability to the effects of climate change. One participant documented: “*I am exposed to quite a lot of climate change factors in my environment, from environmental issues to health issues. On a scale of 1-10, I would say 7*”.

Financial constraint, both at the individual level and for research purposes, was commonly cited as a barrier to the development of adaptation pathways. Many participants observed that addressing climate change necessitates significant funding. One participant stated: “*Inadequate financing is an issue, and the research institutes are poorly funded*". It was also agreed that certain actions to adapt to climate change did not require financial resources. For example, avoiding single-use plastics, refraining from open-air waste burning, and sorting waste to separate recyclable materials before disposal were considered as measures that should be enforced immediately at all levels.

Summarizing the key points, the findings underscored the universal impact of climate change and the necessity for everyone to take collective responsibility in addressing and mitigating its effects. Stakeholders stressed that while policymakers and government entities at all levels play critical roles, it is also important for individuals to embrace and advocate for climate change mitigation on a personal level.

## Discussion

The aims and objectives of the inaugural stakeholders´ engagement meeting were successfully met. The C2Rest project was well-received, and participants actively engaged in the discussions. They shared their experiences and ideas, drawing from their local knowledge of their communities. All stakeholders collectively acknowledged everyone's vulnerability to climate change and the collective responsibility to mitigate its health impacts.

The findings regarding vulnerability to climate change from this stakeholder engagement are consistent with global observations, which identify children, the elderly, and individuals with pre-existing health conditions as the most vulnerable populations to the respiratory health impacts of climate change [13,14]. They also align with the broader literature, which highlights the substantial health risks posed by climate change [2,5,15]. These vulnerable groups often have limited access to healthcare, reside in areas susceptible to flooding, and are more exposed to environmental hazards. This underscores the need to enforce existing environmental regulations and develop new policies that specifically address the health impacts of climate change [13,17].

A significant challenge identified in the study is the sociocultural barrier to effective climate change mitigation. The attribution of climate change to supernatural causes, such as water spirits or acts of God, rather than human activities, reflects additional challenges in climate change communication and education within the African context, where traditional beliefs and misinformation can significantly hinder mitigation efforts [15,18]. Studies have shown that local beliefs and cultural norms can either facilitate or impede climate change adaptation and mitigation [20]. In Nigeria, as in many other African countries, effective climate change communication must consider these cultural contexts to be successful. This involves engaging with local leaders, including religious leaders, and using culturally relevant messages that address misconceptions about climate change. Additionally, community leaders play a vital role in facilitating the execution of climate change mitigation initiatives through multi-sectoral advocacy [14,18]. One of the key recommendations from the stakeholder engagement meeting is the need for capacity building and media engagement to improve population literacy about climate change. Integrating climate change education into the formal education system from kindergarten onwards is a vital initiative. Educating young minds about climate change and its impacts can foster a generation that is more informed and proactive in addressing these challenges. The media must also be well-informed to consistently disseminate well-researched information on climate change and educate the public. Media campaigns should continuously address misinformation and false sociocultural beliefs to improve public understanding and acceptance of climate change mitigation measures [17,20]. Disseminating simplified climate change information in local languages is essential to ensure widespread understanding. Clear, concise communication of research findings tailored to different populations will help bridge the gap between scientific research and public understanding [16,21,22].

This study highlights the need for green initiatives, such as planting trees and establishing green zones in communities. Economic activities should be zoned away from residential areas to reduce exposure to pollutants, and building regulations should promote sustainability by reducing reliance on cement [2]. Shifting towards renewable energy sources, such as solar and wind energy, and adopting cleaner energy alternatives like compressed natural gas (CNG) and liquefied petroleum gas (LPG) are also essential steps in reducing the overall carbon footprint and promoting sustainable development [13,14].

This stakeholder engagement had some limitations, which could have impacted the findings. First, not all participants responded to all questions on the activity list, affecting our understanding of some key issues. The varying levels of engagement and participation could be attributed to several factors, including the complexity of the questions, the participant´s familiarity with the subject, or perhaps the format of the engagement itself. Second, time constraints may have been a limitation. The structure of the meeting, constrained to a single day, may have limited the ability to exhaustively discuss all the questions, particularly those in activity 3, which focused on adaptation pathways. As a result, some valuable insights and discussions may have been shortened or omitted, affecting the comprehensiveness of the responses. Third, not all invited stakeholders attended the engagement meeting. The absence of some key participants meant that some perspectives and expertise were not represented. The missing voices could have provided additional insights or alternative viewpoints that would have enriched the discussion and recommendations. Fourth, the written submissions were anonymized, so we could not create associations based on socio-demographic characteristics. Overall, these limitations highlight the need for ongoing stakeholder engagement. Future meetings could benefit from extended durations to allow longer and likely more in-depth discussions. Strategies to ensure higher attendance and participation from all invited stakeholders must be developed. Addressing these limitations will be crucial for developing a more comprehensive and actionable approach to mitigating the health impacts of climate change in Nigeria.

Despite these limitations, our report had recognized strengths. The diversity of the invited groups, including community and religious leaders, who are often left out in such meetings, provided insight from ordinary non-experts without prior interest in climate change. Using a validated framework to guide the discussion and the informal and participatory format of the meeting encouraged openness.

## Conclusion

The inaugural stakeholders' engagement meeting for the C2Rest Nigeria study was a significant step towards addressing the impacts of climate change on respiratory health in Nigeria. The positive reception of the project and the active participation of stakeholders underscore the importance of a collaborative approach to climate change mitigation. The recommendations from the meeting highlight the need for capacity building, multi-sectoral advocacy, education, and local language dissemination to effectively tackle climate change challenges. While existing policies provide a foundation, there is a critical need for more robust and coordinated actions at all levels of government and society to ensure sustainable, accountable, and transparent climate change mitigation efforts. The inaugural meeting served as a foundational platform for idea generation and collaboration, setting the stage for subsequent qualitative and longitudinal studies to complement the findings of this meeting.
